# Radiographic and magnetic resonance imaging predicts severity of cruciate ligament fiber damage and synovitis in dogs with cranial cruciate ligament rupture

**DOI:** 10.1371/journal.pone.0178086

**Published:** 2017-06-02

**Authors:** Susannah J. Sample, Molly A. Racette, Eric C. Hans, Nicola J. Volstad, Gerianne Holzman, Jason A. Bleedorn, Susan L. Schaefer, Kenneth R. Waller, Zhengling Hao, Walter F. Block, Peter Muir

**Affiliations:** 1 Department of Surgical Sciences, School of Veterinary Medicine, University of Wisconsin-Madison, Madison, Wisconsin, United States of America; 2 UW Veterinary Care Hospital, School of Veterinary Medicine, University of Wisconsin-Madison, Madison, Wisconsin, United States of America; 3 Department of Medical Physics, School of Medicine and Public Health, University of Wisconsin-Madison, Madison, Wisconsin, United States of America; Colorado State University, UNITED STATES

## Abstract

Cruciate ligament rupture (CR) and associated osteoarthritis (OA) is a common condition in dogs. Dogs frequently develop a second contralateral CR. This study tested the hypothesis that the degree of stifle synovitis and cranial cruciate ligament (CrCL) matrix damage in dogs with CR is correlated with non-invasive diagnostic tests, including magnetic resonance (MR) imaging. We conducted a prospective cohort study of 29 client-owned dogs with an unstable stifle due to complete CR and stable contralateral stifle with partial CR. We evaluated correlation of stifle synovitis and CrCL fiber damage with diagnostic tests including bilateral stifle radiographs, 3.0 Tesla MR imaging, and bilateral stifle arthroscopy. Histologic grading and immunohistochemical staining for CD3^+^ T lymphocytes, TRAP^+^ activated macrophages and Factor VIII^+^ blood vessels in bilateral stifle synovial biopsies were also performed. Serum and synovial fluid concentrations of C-reactive protein (CRP) and carboxy-terminal telopeptide of type I collagen (ICTP), and synovial total nucleated cell count were determined. Synovitis was increased in complete CR stifles relative to partial CR stifles (*P*<0.0001), although total nucleated cell count in synovial fluid was increased in partial CR stifles (*P*<0.01). In partial CR stifles, we found that 3D Fast Spin Echo Cube CrCL signal intensity was correlated with histologic synovitis (S_R_ = 0.50, *P*<0.01) and that radiographic OA was correlated with CrCL fiber damage assessed arthroscopically (S_R_ = 0.61, *P*<0.001). Taken together, results of this study show that clinical diagnostic tests predict severity of stifle synovitis and cruciate ligament matrix damage in stable partial CR stifles. These data support use of client-owned dogs with unilateral complete CR and contralateral partial CR as a clinical trial model for investigation of disease-modifying therapy for partial CR.

## Introduction

Cruciate ligament rupture (CR) is an important cause of stifle instability and pelvic limb lameness in dogs [1]. CR is an economically important canine condition in the United States [2]. Mid-substance rupture of the cranial cruciate ligament (CrCL) is not typically associated with contact trauma [1,3]. Ligament matrix fiber damage in the CrCL and the caudal cruciate ligament (CdCL) typical precedes CR [4]. Bilateral CR identified at initial diagnosis or because of subsequent contralateral CR is also common in affected dogs [1,5]. Occasionally, rupture of both the CrCL and the CdCL is identified during surgical treatment of affected dogs. Stifle osteoarthritis (OA) is found at diagnosis in dogs with CR and includes lymphocytic-plasmacytic synovitis [1,6–8]. In dogs with unilateral CR, radiographic signs of synovial effusion and OA are often present in contralateral partial CR stifle joints [1]. Surgical stabilization of the stifle joint is a common treatment for dogs with CR. Functional outcome is procedure-dependent [9,10]. Recent meta-analysis of clinical outcomes most strongly supports use of tibial plateau leveling osteotomy (TPLO) stabilization [11]. The TPLO procedure is not disease-modifying, and worsening synovial effusion and stifle OA is typical over time when unstable stifles are treated with TPLO in affected dogs [12]. TPLO in partial CR stifles may help to better protect the CrCL from progressive fiber tearing and ultimately reduce disease progression [13].

The CR condition is progressive and degenerative. Stifle synovitis develops in the early phase of the disease before development of joint instability [6,7]. Joint instability results from cruciate ligament sprain. Ligament sprains are defined biomechanically by structural damage and the degree of associated joint laxity. Grade I sprains are associated with mild fiber damage and no joint laxity. Grade II sprains are associated with moderate fiber damage and a stretch to the point of detectable joint laxity. Grade III sprains are associated with severe disruption of ligament fibers and obvious joint laxity [14]. Consequently, an unstable stifle is the consequence of complete CR. Stifle joints with partial CR are stable with ligament fiber rupture evident with arthroscopic examination [6]. Partial CR stifles have radiographic signs of synovial effusion and stifle OA, and may have clinically detectable periarticular fibrosis [1,5,6]. Even mild radiographic change appears clinically relevant [1,5]. Lameness is often associated with partial CR in dogs [13,15]. In dogs with unilateral complete CR, the presence of stifle synovial effusion and osteophytosis in the contralateral stable partial CR stifle is predictive of risk for contralateral complete CR [1,3,5]. Histological synovitis in complete CR stifles is also predictive of risk of subsequent contralateral complete CR in dogs [16].

Clinically relevant diagnostic markers that reflect severity of cruciate ligament fiber damage and stifle synovitis would be valuable tools to improve management of affected dogs. Early identification of partial CR dogs at high risk of complete CR could facilitate implementation of disease-modifying medical or surgical treatment [3,13,17]. Similarly, in dogs with unilateral complete CR, such markers could be used to predict risk of subsequent contralateral rupture, such that this risk could be considered in patient management.

In addition to radiography, which can easily be performed clinically, 3.0 Tesla MR imaging is a promising tool that provides superior imaging of stifle soft tissue structures. MR imaging reflects normal CrCL structural properties [18–20]. The relationship between MR signal changes and ligament fiber damage in dogs with partial CR is unclear, although signal changes in ligament attachment sites and subchondral bone are commonly found in affected dogs [21,22]. MR imaging can also be used to assess stifle synovium [21]. Joint capsule consists of an outer fibrous layer and an inner synovial membrane, which lines all structures in the stifle and contains an extensive network of blood and lymphatic vessels and nerves. In dogs with CR, macroscopic changes in the stifle synovium, visible arthroscopically, include villus hypertrophy and increases in the density, size and tortuosity of synovial blood vessels [6,7]. CrCL structural properties are significantly reduced by stifle synovitis [23], suggesting that inflammatory changes within stifle synovial membrane are important factors influencing risk of CR, at least with regards to disease progression through progressive fiber damage and eventual development of a mid-substance rupture in the absence of contract trauma [1].

The present study aimed to evaluate correlations between radiographic, MR imaging, arthroscopic, histologic and cytologic findings in the stifle joints of dogs with unilateral complete CR and contralateral partial CR [6]. We hypothesized that MR imaging and quantification of relevant biomarkers would correlate with severity of CrCL fiber damage in stifles with partial CR and synovitis in both partial and complete CR stifles. Confirmation that MR imaging and biomarker quantification reflect CrCL fiber damage in dogs with partial CR could be relevant to clinical management of affected dogs. We designed this study to provide a foundation for, and inform design of, future longitudinal studies evaluating disease-modifying treatment for canine CR.

## Materials and methods

### Dogs

**Fig 1:**
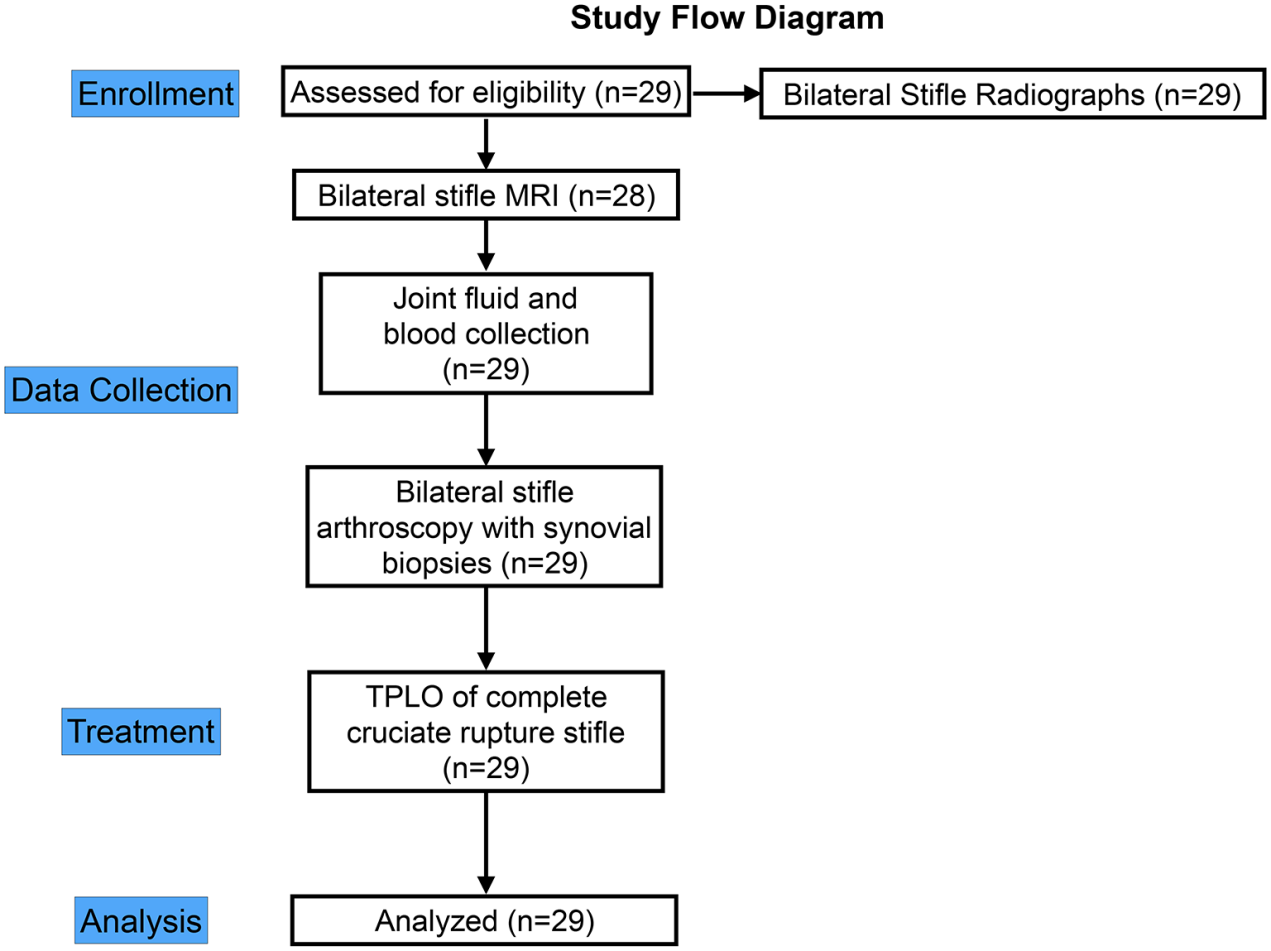
Study design flow diagram illustrating enrollment, data collection, treatment and analysis. TPLO—tibial plateau leveling osteotomy for treatment of stifle instability from CR.

Twenty-nine dogs with unilateral complete CR and a contralateral partial CR were prospectively recruited at the University of Wisconsin-Madison UW Veterinary Care Hospital for a clinical trial between April 2013 and June 2014 (Fig 1). Passive stifle stability was assessed with cranial drawer and cranial tibial thrust tests with both flexion and extension of the stifle under sedation [24] and repeated under general anesthesia. Inclusion criteria were: (1) clinical signs of unilateral pelvic limb lameness; (2) a unilateral complete CR as determined by palpable cranial translation of the tibia relative to the femur; (3) a contralateral stifle joint with no evidence of Grade III sprain (no obvious cranial drawer or cranial tibial thrust in contralateral stifle under sedation); 4) radiographic evidence of bilateral stifle synovial effusion and osteophytosis [7]. Dogs were excluded if history and physical examination suggested traumatic injury, if other stifle pathology was present, or if there was history of previous stifle surgery. Age, weight, gender, and history were recorded for each dog. After diagnosis, the complete CR stifle was treated using TPLO.

### Ethics statement

All procedures at the University of Wisconsin-Madison were conducted with the approval of the Animal Care & Use Committee, School of Veterinary Medicine, University of Wisconsin-Madison (V1070). Dogs were recruited with informed written consent from each owner.

### Radiography

Initially, weight-bearing lateral medial stifle radiographs were made bilaterally to determine the functional length of the CrCL under load [25]. Orthogonal cranial caudal and medial lateral radiographic views of both stifles were subsequently obtained under sedation with the stifle flexed to ninety degrees. Radiographic effusion and radiographic OA were used to evaluate the severity of stifle inflammation [1,8]. The most important early radiographic sign of stifle OA in dogs with CR is development of synovial effusion and associated compression of the infrapatellar fat pad [1,5]. Radiographic effusion was graded subjectively on a scale from 0–2 (0—normal, 1—mild, 2—severe) [1]. Both cranial and caudal stifle joint spaces were considered in this grading. Cranially, the extent of effusion and the shape of the infrapatellar fat pad were considered. Caudal bulging of the joint capsule was also evaluated. Radiographic OA was graded subjectively on a scale from 0–3 (0—normal, 1—mild, 2—moderate, 3—severe) based on the severity of osteophytosis at the margins of the stifle joint [1]. In addition, the tibial plateau angle (TPA) was calculated for each stifle from the lateral radiographic views of the tibia, with the stifle and tarsus held in ninety degrees of flexion. The functional length of the CrCL was determined from the distance between the femoral and tibial attachments on weight-bearing lateral radiographs [25], and was normalized to patellar length (CrCL_d_) to account for variation in dog size. Radiographic scoring was performed by a single observer (PM).

### Magnetic resonance imaging

A 3.0 Tesla MR imaging scanner (GE 3T 750 Discovery, GE Healthcare, Waukesha, WI) and a Neocoil 16 Channel Medium Flex Coil was used. All MR imaging sequences were obtained under general anesthesia. 3D fast spin echo (FSE) Cube sequences were obtained from both stifles. For the partial CR stifle, Vastly under-sampled Isotropic PRojection with alternating length repetition times (VIPR-aTR) sequences, and pre- and post-contrast sagittal T1-weighted sequences were also obtained. MR sequence specifications for the 3D FSE Cube were TR– 1600ms, TE– 22.0ms, flip angle– 90 degrees, and an image matrix size of 384 x 384 x 60 over a 140 x 140 x 60mm field-of-view for a resolution (voxel dimension) of 0.36 x 36 x 1mm. For the VIPR-aTR sequence, specifications were TR– 4.6ms and flip angle– 15 degrees. Two radial lines are acquired per TR in an out and back trajectory over 3.6ms echo times of 0.5 and 3.2ms. VIPR-aTR has an image matrix size of 320 x 320 x 320 over a 120 x 120 x 120mm field-of-view for a 0.375 x 0.375 x 0.375mm voxel dimension. The sagittal T1-weighted sequence was obtained before and after intravenous injection of gadolinium (50–60 mg/kg, MultiHance, Bracco Diagnostics Inc., NJ). MR sequence specifications for the T1-weighted sequence were TR– 700ms, TE– 10.9ms, flip angle– 90–111 degrees, and an image matrix size of 384 x 224mm over a 160 x 160mm field-of-view for a resolution (voxel dimension) of 0.41 x 0.71mm.

Sequences were chosen and oriented to optimize imaging of the cruciate ligament complex. Measurements of CrCL volume and median grayscale value in the 3D FSE Cube and VIPR-aTR sequences were made using 3D segmentation software (Mimics, Materialise, Belgium). 3D FSE Cube and VIPR-aTR volume measurements were normalized to radiographic patella length (CrCL FSE Volume, CrCL VIPR Volume, respectively). The median grayscale values for the 3D FSE Cube and VIPR-aTR sequences were normalized to the grayscale value of the cranial tibial muscle, obtained from a single slice (CrCL FSE Grayscale, CrCL VIPR Grayscale, respectively). The cranial tibial muscle was selected for normalization because its large volume in the image field would minimize background signal variation [20]. The difference between the median grayscale values before and after contrast injection, both of which were normalized to the cranial tibial muscle median grayscale value pre-contrast, was also determined from T1-weighted sequences to estimate CrCL ligament contrast enhancement (CrCL T1 Enhance). Measurements were made by a single observer (NV).

### Arthroscopy

Before TPLO, both stifles were examined using a 2.7 or 2.9 mm 30° rigid arthroscope placed in a medial parapatellar mini-arthrotomy incision. Joint regions (lateral and medial pouches, lateral and medial femoro-tibial joint compartments, the intercondylar notch, and the femoro-patellar joint) were evaluated [7]. Severity of synovitis and cruciate ligament fiber damage were graded using a standardized scoring system [7]. Three parameters describing macroscopic inflammation were evaluated for each joint region: synovial hypertrophy, vascularity, and synovitis [7]. A total arthroscopic synovitis score was calculated as the sum of each parameter’s grade [7,26] (Total Arthroscopic Score). Additionally, the degree of global synovitis was scored using a visual analog scale (VAS) (0–100) for each stifle joint, with 0 representing no inflammation, and 100 signifying the most severe inflammation (Arthroscopic Synovitis VAS) [7]. The CrCL and the CdCL were inspected and probed for evidence of pathologic change, including fiber rupture, color change, and hypertrophy of ruptured ends of ligament fascicles. The extent of fiber disruption was estimated using a calibrated arthroscopic probe. The degree of CrCL fiber tearing in the partial CR stifle (Arthroscopic CrCL Fiber Damage VAS) was determined using a visual analog scale (0–100), with 0 representing no damage, and 100 signifying complete ligament fiber rupture. The lateral and medial menisci were also inspected and probed for tears. A biopsy of synovial membrane was collected from the medial joint pouch of each stifle after completion of arthroscopic observation.

### Histology

Immediately after collection, synovial biopsies were fixed in Zamboni’s fixative for 1–2 days at 4°C [27]. Multiple paraffin-embedded sections, 10μm thick, were prepared. Sections were stained with hematoxylin and eosin (H&E) stain for assessment of cellular infiltration. Additional slides were deparaffinized [28] for immunohistochemical and histochemical staining [7,29].

For Factor VIII immunohistochemical staining of blood vessels, slides were treated with 0.05% pronase in phosphate buffered saline (PBS) for 10 minutes at 37°C and 0.01% trypsin in 0.1% calcium chloride for 20 minutes at 37°C in a humidified chamber. Sections were cooled to room temperature for 10 minutes and then rinsed in PBS twice for 5 minutes. Sections were treated with 3% H_2_O_2_ in PBS for 10 minutes to block endogenous peroxidase activity and then rinsed in PBS. Sections were blocked with 10% normal goat serum in PBS for 30 minutes to block non-specific binding of secondary immunoglobulins. Primary antibody staining used a dog cross-reactive polyclonal rabbit anti-human Factor VIII (#A0082 Dako, Carpinteria, CA), diluted 1:500 in PBS at room temperature for 1 hour. Sections were then rinsed in PBS three times for 5 minutes each. The secondary antibody staining used was a biotinylated goat anti-rabbit IgG (#BA-1000, Vector, Burlingame, CA) with HRP-streptavidin (#434323, Invitrogen, San Francisco, CA) reagent diluted 1:500 in PBS for 30 minutes at room temperature. Positive staining was identified using the 3-amino-9-ethylcarbazole chromogen (#00–1111 Invitrogen, San Francisco, CA). Slides were counterstained with Mayer’s hematoxylin. Negative controls were prepared without the primary antibody or without the secondary antibody to validate the staining.

For CD3 immunohistochemical staining, slides were treated with 10 mMol/L sodium citrate buffer (pH 6.0) at 95–100°C for 30 minutes, cooled to room temperature, and then rinsed in PBS twice for 5 minutes. Sections were treated with 3% H_2_O_2_ in PBS for 10 minutes to block endogenous peroxidase activity and rinsed in PBS. Sections were then treated with 10% normal goat serum in PBS for 30 minutes to block non-specific binding of secondary immunoglobulins. Primary antibody staining used a rat anti-human immunoglobulin (#MCA1477T, Serotec, Raleigh, NC) that is cross-reactive with the dog, diluted 1:500 in PBS overnight at 4°C. Sections were then rinsed in PBS three times for 5 minutes each. Secondary antibody staining used a biotinylated goat anti-rat IgG (#BA-9400, Vector, Burlingame, CA) with HRP-streptavidin reagent (#434323, Invitrogen, San Francisco, CA) diluted 1:500 in PBS for 30 minutes each at room temperature. Sections of dog lymph node were used as a positive control. The chromogen, counterstain, and negative controls were prepared as for Factor VIII staining.

For histochemical staining of TRAP, a solution of naphthol AS-BI phosphate was prepared by dissolving 25mg of naphthol AS-BI phosphate in 2.5ml of n,ndimethylformamide to which 45ml of 0.05M Trismaleate buffer (pH 5.0) was added. A solution of hexazotized pararosanaline was prepared by dissolving 0.25g of pararosaniline hydroxychloride in 5mL of distilled water, to which 1.25mL of hydroxychloric acid was added. This solution was mixed with an equal volume of 4% sodium nitrite immediately before use. The final reaction mixture for histochemical staining was prepared by adding 4ml of hexazotized pararosanaline solution to the naphthol AS-BI phosphate solution, together with 50mM sodium-potassium tartrate. The final reaction mixture was filtered before use. Sections were incubated in the reaction mixture at 37°C for 1 to 2 hours, rinsed in distilled water, counterstained in Mayer’s hematoxylin, and mounted. Negative controls were performed without the naphthol AS-BI phosphate to validate the protocol.

### Histomorphometry

For H&E stained slides, lymphocytic-plasmacytic and suppurative inflammation of the synovial intima, synovial cell hypertrophy, and synovial intima width, seen at the center of five random high power fields, were subjectively graded using numerical rating scales by a single grader (PM) [26]. Lymphocytic-plasmacytic inflammation was graded from 0 to 3 using the following scale: 0—no inflammation, 1 –infiltration without alteration of tissue architecture, mostly perivascular or sub-intimal, 2 –infiltration with occasional alteration of tissue architecture, numerous inflammatory cells, 3 –infiltration with extensively obscured tissue architecture, dense inflammatory infiltrate and/or follicle formation. Suppurative inflammation was graded as: 0—no inflammation, 1 –negligible inflammation; 2 –present. Synoviocyte cell hypertrophy was graded as follows: 0—normal, 1—mild, 2 –moderate or focal severe, 3 –severe. Synovial line cell layer width, seen at the center of five random high power fields, was measured to determine lining layer depth of each biopsy sample [30] and graded as follows: 0 –one cell layer, 1–2–3 cell layers, 2–4–5 cell layers, 3 –>5 cell layers. A synovial pathology score for each stifle was calculated as the sum of the scores assigned to lymphocytic-plasmacytic inflammation, synoviocyte cell hypertrophy and synovial line cell layer thickness. A synovial pathology grade (Histologic Synovitis Grade) was assigned based on the synovial score (grade 0/no synovitis = sum 0–1; grade 1/low-grade synovitis = sum 2–4; grade 2/high-grade synovitis = sum 5–9) [26]. Additionally, a visual analog scale score for overall synovitis (Histologic Synovitis VAS) was determined using a 100mm horizontal line, the end-points of which were set by the descriptor of no inflammation (0) and most severe inflammation (100).

Evaluation of histochemical and immunohistochemical staining also used a single individual grader (SJS). For CD3^+^ T lymphocytes (CD3^+^ T Lymphocyte Grade), synovial biopsies were graded on a numeric scale based on the amount of CD3^+^ T lymphocytes seen according to the following grades: 0 –no positive cells, 1 –occasional scattered cells, 2 –mild infiltration, 3 –moderate to severe infiltration, 4 –score of 3 with additional of lymphoid nodule formation. A similar grading scale was used for TRAP^+^ macrophages (TRAP^+^ Macrophage Grade), as follows: 0 –no positive cells, 1 –rare occasional scattered cells (<5 cells/slide), 2 –occasional cells or mild focal inflammation, 3 –mild generalized infiltration or focal moderate to severe inflammation, 4 –moderate to severe generalized infiltration. Synovial biopsies were also evaluated for factor VIII^+^ vessels (Factor VIII^+^ Vessel Grade) using the following grade system: 0 –no positive uptake within sectioned sample, 1 –mildly elevated vessel density, 2 –moderate elevated vessel density, 3 –markedly elevated vessel density. Additionally, a visual analog scale score for Factor VIII^+^ vessel staining (Factor VIII^+^ Vessel VAS) was determined using a 100mm horizontal line, the end-points of which were set by the descriptor of no positive stain (0) and most severe stain uptake (100).

### Synovial and serum markers of inflammation and ligament matrix degradation

Synovial fluid total nuclear cell count (TNCC) in complete CR and partial CR stifles was estimated using direct smears of synovial fluid obtained by arthrocentesis and stained with Wright-Giemsa stain. Quality control for direct smears was as described [31]. Manual counting of synovial cells on each smear was performed using a validated estimation method [31]. All nucleated cells in a field were counted, including those with pyknotic and karyorrhectic nuclei, cells forming groups, and naked nuclei. A cell was considered within the counting field if at least half the cell was within the microscopic field. Nucleated cells in fifteen 400x fields were counted and the mean number of nucleated cells per 400x field was determined. After correction for the dimensions of the 400x field area, TNCC was estimated (Stifle TNCC) using the regression formula (y = 0.45x-0.36) [31].

C-Reactive Protein (CRP) and pyridinoline cross-lined carboxy-terminal telopeptide of type I collagen (ICTP) were quantified in serum and synovial fluid (Serum CRP, Serum ICTP, Synovial CRP, Synovial ICTP) using commercial canine-specific ELISAs (CRP—Immunology Consultants Laboratory, Ict., Portland, OR; ICTP—NeoScientific, Cambridge, MA). Synovial fluid/serum CRP and ICTP ratios were also calculated (Synovial:Serum CRP, Synovial:Serum ICTP).

### Statistical analysis

For both complete and partial CR stifles, Histologic Synovitis Grade and Histologic Synovitis VAS were used as the comparator for other variables. For partial CR stifles, Arthroscopic CrCL Fiber Damage VAS was also a comparator. Other variables included radiographic, MR imaging, histologic and biochemical variables (S1 Table). Radiographic variables included Radiographic Effusion, Radiographic OA, and CrCL_d_. MR imaging variables evaluated for both stifles included CrCL FSE Volume and CrCL FSE Grayscale. For the partial CR stifle, CrCL VIPR Volume, CrCL VIPR Grayscale and CrCL T1 Enhance were also included as MR imaging variables. Arthroscopic variables for both stifles included Total Arthroscopic Score and Arthroscopic Synovitis VAS. For the partial CR stifle, Arthroscopic CrCL Fiber Damage VAS was also included. Histologic grading of synovial biopsies included Histologic Synovitis VAS, CD3^+^ T Lymphocyte Grade, TRAP^+^ Macrophage Grade, Factor VIII^+^ Vessel Grade, and Factor VIII^+^ Vessel VAS. Biochemical markers included Serum CRP, Serum ICTP, Synovial CRP, Synovial ICTP, Synovial:Serum CRP, Synovial:Serum ICTP and Stifle TNCC.

Data were reported as mean ± standard error or median (range) as appropriate for parametric or non-parametric data, respectively. A Shapiro-Wilk’s test was used to determine if data approximated a normal distribution for continuous variables. The Spearman Rank correlation test was used to determine correlation between potentially predictive variables at diagnosis with both histologic synovitis grade and histologic synovitis VAS for each stifle and arthroscopic CrCL fiber damage in the partial CR stifle. Stifles were treated as separate experiments for correlative analysis, as disturbances to biological pathways in the early and late phases of the disease may be different. For comparison of variables between stifles, a paired Students T-test or a Wilcoxon matched-pairs signed rank test, for parametric or non-parametric data, respectively, was used. Spearman Correlation plots were drawn using R version 3.1.3 (http://www.r-project.org/) to examine the interrelationship between markers for the complete and partial CR stifles. Ward’s method for hierarchical clustering was used, with addrect set to 3 or 4. Results were considered significant at *P≤*0.05. Trends were also reported (*P≤*0.1).

## Results

### Clinical findings

Twenty nine dogs were enrolled (Fig 1). The age of the group was 5.5±0.5 years old (range: 1.6 to 9.9 years). Body weight was 37.1±1.7kg (range: 24.1 to 59.0kg). There were 3 males, 12 castrated males, and 14 ovariohysterectomized females. A range of breeds was represented, and included 11 Labrador Retrievers, 6 mixed breed dogs, 3 Golden Retrievers, 2 Boxers, and one each of the following: German Shorthair Pointer, German Shepherd Dog, French Mastiff, Dalmatian, Springer Spaniel, Rottweiler, and Newfoundland. Nineteen dogs had left complete CR and 10 dogs had right complete CR. At entry, 17 dogs were reported to receive non-steroidal anti-inflammatory (NSAID) medication on a regular basis, and 9 dogs were not receiving NSAID medication. Owners of 3 dogs were unsure whether their dog had received NSAID treatment. All dogs had unilateral palpable cranial translation of the tibia relative to the femur on sedated exam, indicative of complete CR. Dogs had no evidence of cranial drawer or cranial tibial thrust in the contralateral stable CR stifle under sedation, and did have radiographic evidence of joint effusion and/or OA, suggesting partial CR (Grade I sprain). Under general anesthesia, four dogs were noted to have evidence of a Grade II sprain in the partial CR stifle. Two dogs had a subtle cranial drawer of the partial CR stifle with <3mm of translation when the stifle was placed in flexion, but did not have cranial drawer when the stifle was in extension. A third dog had subtle mild cranial drawer with <3mm of translation of the partial CR stifle in both flexion and extension. A fourth dog had subtle cranial drawer with <3mm of tibial translation of the tibia and also had a <3mm of positive tibial thrust test in the partial CR stifle.

### Radiography, MR imaging and arthroscopy

**Fig 2:**
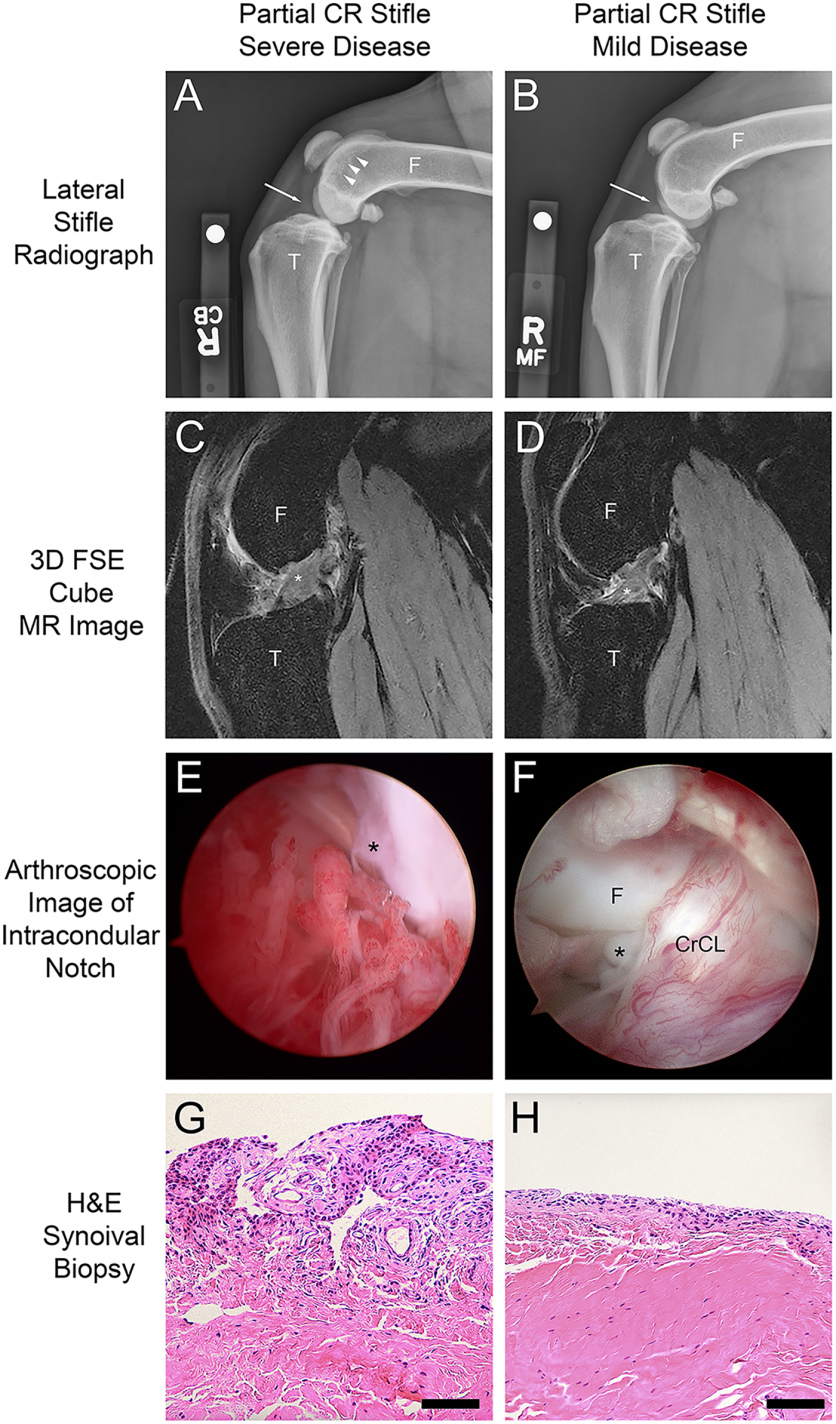
Clinical and pathological features of partial cruciate rupture (CR) in the dog. (A,B) Radiographic features from dogs with severe and mild disease in partial CR stifles. Synovial effusion (white arrows), and osteophyte formation (white arrow heads) is more evident in the stifle with more severe inflammatory synovitis pathology (A) (Radiographic effusion score = 1; Radiographic osteoarthritis (OA) score = 2) than the stifle with more mild disease (B) (Radiographic effusion score = 0; Radiographic OA score = 0). The radiographic scores for the mildly affected stifle were given a score of 0 by the radiographic grader (PM), but the attending clinician evaluating the case for study inclusion considered the radiographs abnormal at diagnosis. (C,D) Sagittal plane 3D FSE Cube MR images of the cranial cruciate ligament (CrCL) (white asterisk) shows that the CrCL from the partial CR stifle with more severe disease (C) is thickened compared to the partial CR stifle with more mild disease (D). (E,F) Arthroscopic images obtained from the intercondylar notch highlight the more prominent vascularity, hypertrophy and synovitis in the stifle with more severe disease (E) (Total Arthroscopic Score = 23) than the stifle with more mild disease (F) (Total Arthroscopic Score = 19). (G,H) Torn CrCL fibers are evident in both images (black asterisk). Biopsies of stifle synovial lining obtained from the medial joint compartment show the stifle with more severe disease (G) (Total Histologic Grade = 2; Histologic VAS score = 96) has greater inflammatory infiltrate, synovial cell hypertrophy and synovial intima thickness when compared to the partial CR stifle with mild disease (H) (Total Histologic Score = 1; Histologic VAS score = 6). Scale bar = 0.5mm.

Data are summarized in Table 1. Grades for radiographic effusion and OA were increased in the complete CR stifle, compared to the partial CR stifle (*P*<0.0001, Fig 2, Table 1). CrCL_D_ was increased in the complete CR stifle, compared to the partial CR stifle (*P*<0.0001, Table 1). TPA in the complete and partial CR stifles ranged from 20 to 31 degrees and 21 to 26 degrees, respectively, and was increased in the complete CR stifle, compared to the partial CR stifle (*P* = 0.04, Table 1). MR imaging was performed in 28 of the 29 dogs (Fig 1). 3D FSE Cube sequences were obtained bilaterally from 28 dogs (Fig 2). VIPR-aTR sequences of the partial CR stifle were obtained from 20 of 29 dogs. CrCL T1 Enhance was determined in the partial CR stifle in 28 dogs. CrCL FSE Volume and CrCL FSE Grayscale measurements were respectively decreased and increased in the complete CR stifle, compared to the partial CR stifle (*P*<0.0001, *P* = 0.04, respectively, Table 1). The four dogs with mild instability of the partial CR stifle noted under general anesthesia, indicative of Grade II CrCL sprain, did not have partial CR CrCL ligament volumes that deviated from dogs without observed instability. During arthroscopy, torn CrCL fibers were found in all partial CR stifles (Fig 2). Medial meniscus tears were found in 20 of 29 complete CR stifles. No tears were identified in the lateral meniscus in either stifle joint, and no meniscal tears were noted in the partial CR stifles. Synovitis Score and Synovitis VAS score were increased in the complete CR stifle compared to the partial CR stifle (*P*<0.0001, Table 1).

**Table 1:** Radiographic, magnetic resonance imaging and arthroscopic findings.

Parameter	Complete CR stifle	Partial CR stifle	*P*-value
**Synovial Effusion**			
Grade 0	0 (0%)	2 (7%)	n/a
Grade 1	1 (3%)	17 (59%)	n/a
Grade 2	28 (97%)	10 (34%)	n/a
**Osteophytosis**			
Grade 0	0 (0%)	5 (17%)	n/a
Grade 1	3 (10%)	12 (41%)	n/a
Grade 2	6 (21%)	0 (0%)	n/a
Grade 3	20 (69%)	2 (7%)	n/a
**Morphometric parameters**			
CrCL_D_	1.53±0.03	1.32±0.02	<0.0001
TPA	27.07±0.53	26.24±0.61	0.04
**MR imaging quantification**			
CrCL FSE volume (mm^3^/mm)	9.63±0.61	24.35±1.25	<0.0001
CrCL VIPR volume (mm^3^/mm)	n/a	23.57±1.37	n/a
CrCL FSE Grayscale^c^	1.01±0.06	0.84±0.05	0.04
CrCL VIPR Grayscale^c^	n/a	0.89±0.06	n/a
CrCL T1 Enhance	n/a	0.10±0.07	n/a
**Arthroscopy**			
Synovitis Score	46.17±1.56	32.10±1.96	<0.0001
Synovitis VAS	66.14±2.91	35.2±3.51	<0.0001
Fiber Damage VAS	n/a	25.21±3.69	n/a

**Note:** *P*-values represent differences in complete and partial CR stifle pairs. n = 28–29 for all correlations except for those pertaining to partial CR stifle VIPR imaging where n = 20. **Abbreviations**: CR, cruciate rupture; SD, standard deviation; VAS, visual analogue scale score; CrCL, cranial cruciate ligament; FSE, fast spin echo; VIPR, Vastly under-sampled Isotropic Projection; n/a, not applicable.

^c^Ligament grayscale value was normalized to the cranial tibial muscle grayscale value.

### Inflammation in synovium, synovial fluid and serum

Results are summarized in Table 2. Histologic Synovitis Grade consisted of Grade 1 in 9 and Grade 2 in 20 complete CR stifles; 2 partial CR stifles were grade 0, 11 were Grade 1 and 16 were Grade 2. Crush artifact prevented analysis of some sections for TRAP^+^ (n = 1), and Factor VIII^+^ (n = 2) staining. TRAP^+^ Macrophage Grade and Factor VIII^+^ Vessel VAS score were increased in the complete CR stifle, compared to the partial CR stifle (*P* = 0.04, *P* = 0.01, respectively, Table 2). Serum CRP and ICTP concentrations at diagnosis were 4784 (837–113721) and 1.67 (0.42–11.58) μg/L, respectively. Synovial CRP and Synovial:Serum CRP were increased in complete CR stifles, compared to partial CR stifles (*P* = 0.001, *P* = 0.0001, respectively, Table 2). Synovial TNCC was increased in partial CR stifles compared to complete CR stifles (*P* = 0.01, Table 2).

**Table 2:** Inflammation in stifle synovium and stifle synovial fluid.

Parameter	Complete CR stifle	Partial CR stifle	*P*-value
Histologic Synovitis Grade	2 (1–2)	2 (0–2)	0.21
Histologic Synovitis VAS	58.59±3.72	50.52±5.04	0.11
CD3^+^ T Lymphocyte Grade	1 (0–2)	1 (0–2)	0.15
TRAP^+^ Macrophage Grade	1 (0–3)	1 (0–3)	0.04
Factor VIII^+^ Vessel Grade	2 (1–3)	2 (1–3)	0.15
Factor VIII^+^ Vessel VAS	49.29±3.28	38.85±3.35	0.01
Synovial CRP (μg/L)	1789 (142–10,471)	1345 (48–18,265)	0.001
Synovial:Serum CRP	0.34 (0.06–0.85)	0.17 (0.03–0.97)	0.0001
Synovial ICTP (μg/L)	3.67 (2.22–11.99)	4.18 (2.15–6.25)	0.81
Synovial:Serum ICTP	2.37 (0.40–7.43)	2.73 (0.38–7.43)	0.55
Synovial TNCC (x1000/μL)	0.39 (0–2.17)	1.19 (0–22.89)	0.01

**Note:** *P*-values represent differences in complete and partial CR stifle pairs. Data represent mean±standard error or median (range). n = 26–29 dogs. **Abbreviations**: TRAP, tartrate-resistant acid phosphatase, a marker of activated macrophages; VAS, visual analogue scale score; CRP, C-reactive protein, an acute phase protein; ICTP, pyridinoline cross-lined carboxy-terminal telopeptide of type I collagen, a marker of type I collagen degradation.

### Correlative analysis

#### Correlations with Histologic Synovitis Grade

**Fig 3:**
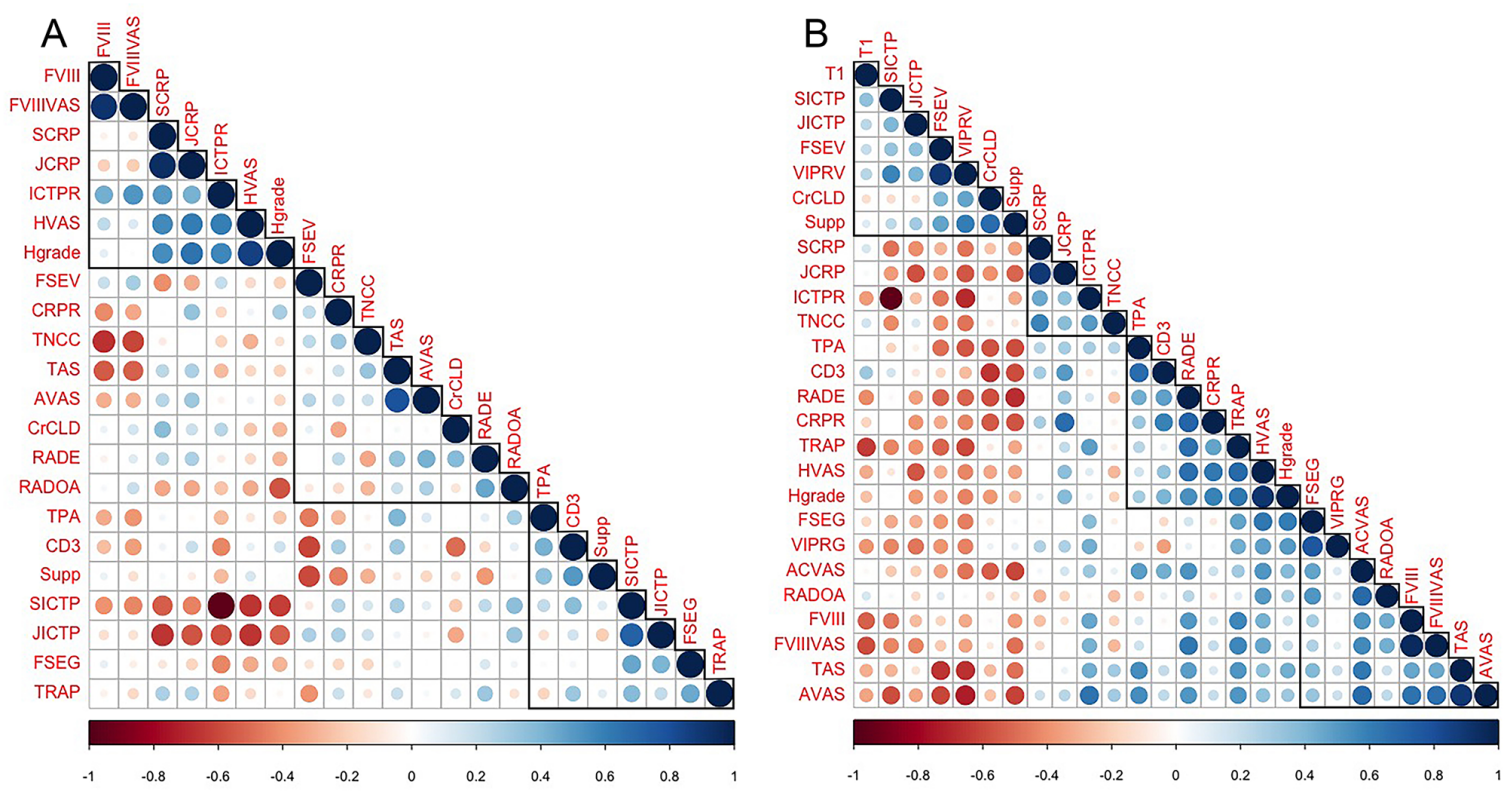
Relationships between diagnostic variables in complete and partial cruciate rupture stifles to evaluate patterns of correlation between markers. (A) In the complete CR stifle, correlations formed three clusters. Several inflammation markers were positively correlated with Synovial and Serum CRP concentrations, suggesting that inflammation promotes collagen degradation within affected stifles. Serum CRP was also positively correlated with histologic inflammation. In a second cluster, Radiographic effusion and OA were positively correlated with arthroscopic synovitis variables. In a third cluster, numbers of CD3^+^ lymphocytes were positively correlated with numbers of TRAP^+^ macrophages and neutrophils. (B) In the partial CR stifle, a larger number of positive correlations were identified that formed four clusters. Suppurative inflammation was positively correlated with CrCL ligament volume, assessed by MR imaging, and functional length of the ligament, suggesting that acute inflammation is related to ligament edema and loss of mechanical properties. In a second cluster, synovial and serum CRP concentrations were correlated with stifle TNCC, indicating that biochemical markers of inflammation correlate with inflammatory cell counts. In a third cluster, the synovial to serum CRP ratio was positively correlated several histologic markers of inflammation, suggesting that the synovial to serum CRP ratio is likely a clinically useful marker of stifle synovitis. In a fourth cluster, arthroscopic variables of inflammation were correlated with MR imaging measures of ligament fluid content, as measured by grayscale value, suggesting that early in the CR condition, synovitis may result in increased ligament fluid content. **Abbreviations**: **TAS**, Total Arthroscopic Score; **ACVAS**, Arthroscopic CrCL Fiber Damage Visual Analog Scale (VAS) score; **AVAS**, Arthroscopic Synovitis VAS score; **CD3**, CD3^+^ T Lymphocyte Grade; **CrCLD**, Radiographic length of CrCL normalized to patellar length; **CRPR**, C-reactive Protein (CRP) serum to synovial fluid ratio; **FSEG**, MR imaging CrCL FSE Grayscale; **FSEV**, MR imaging CrCL FSE Volume; **FVIII**, Synovial Factor VIII^+^ Vessel Grade; **FVIIIVAS**, Synovial Factor VIII^+^ Vessel VAS; **Hgrade**, Histologic Synovitis Grade; **HVAS**, Histologic Synovitis VAS Score; **ICTPR**, pyridinoline cross-lined carboxy-terminal telopeptide of type I collagen (ICTP) serum to synovial fluid ratio; **JCRP**, Synovial fluid CRP; **JICTP**, Synovial fluid ICTP; **RADE**, Radiographic Effusion score; **RADOA**, Radiographic OA score; **SCRP**, Serum C-Reactive Protein; **SICTP**, Serum ICTP; **Supp**, Suppurative Inflammation Grade; **T1**, MR imaging CrCL T1 Enhancement; **TNCC**, Synovial fluid total nucleated cell count; **TPA**, Tibial Plateau Angle; **TRAP**, TRAP^+^ Macrophage Grade; **VIPRV**, MR imaging CrCL VIPR Volume; **VIPRG**, MR imaging CrCL VIPR Grayscale.

Correlation analysis is presented in Tables 3 to 7 and Fig 3. For complete CR stifles (Fig 3A), the following factors were significantly correlated with Histologic Synovitis Grade: Histologic Synovitis VAS (S_R_ = 0.62, *P* = 0.0003) and Synovial CRP (S_R_ = 0.44, *P* = 0.02). Histologic Synovitis Grade was weakly correlated with the following factors: Serum CRP (S_R_ = 0.32, *P* = 0.09), Serum ICTP (S_R_ = -0.36, *P* = 0.06), and Synovial:Serum ICTP (S_R_ = 0.36, *P* = 0.06).

**Table 3:** Correlation between radiographic measures, Histologic Synovitis Grade and cranial cruciate ligament fiber damage.

	Radiographic Effusion		Radiographic OA		CrCL_D_	
	S_R_	*P value*	S_R_	*P value*	S_R_	*P value*
**Complete CR Stifle**						
**Histologic Synovitis Grade**	-0.13	0.51	-0.30	0.11	-0.20	0.31
**Histologic Synovitis VAS**	0.02	0.91	0.02	0.93	-0.13	0.51
**Partial CR Stifle**						
**Histologic Synovitis Grade**	0.30	0.12	0.27	0.15	-0.08	0.64
**Histologic Synovitis VAS**	*0*.*38*	*0*.*04*	*0*.*47*	*0*.*01*	-0.12	0.54
**Arthroscopic Fiber Damage VAS**	0.30	0.11	*0*.*61*	*0*.*0005*	-0.30	0.11

**Note**: Arthroscopic fiber damage VAS assesses ligament fiber rupture in the cranial cruciate ligament in the partial CR stifle. n = 29 dogs. **Abbreviations**: CR, cruciate ligament rupture; CrCL, cranial cruciate ligament; CrCL_D_, radiographic length of the CrCL normalized to patellar length.

**Table 4:** Correlation between histologic synovitis and arthroscopic assessment of synovitis and cranial cruciate ligament fiber damage.

	Arthroscopic Synovitis Score		Arthroscopic Synovitis VAS		Arthroscopic Fiber Damage VAS	
	S_R_	*P value*	S_R_	*P value*	S_R_	*P value*
**Complete CR Stifle**						
**Histologic Synovitis Grade**	0.01	0.95	-0.09	0.65	n/a	
**Histologic Synovitis VAS**	0.02	0.91	0.21	0.27	n/a	
**Partial CR Stifle**						
**Histologic Synovitis Grade**	*0*.*43*	*0*.*02*	0.23	0.22	0.24	0.21
**Histologic Synovitis VAS**	0.20	0.29	0.22	0.26	*0*.*38*	*0*.*04*
**Arthroscopic Fiber Damage VAS**	*0*.*41*	*0*.*03*	*0*.*46*	*0*.*01*	n/a	

**Note**: Arthroscopic fiber damage VAS assesses ligament fiber rupture in the cranial cruciate ligament in the partial CR stifle. n = 29 dogs. **Abbreviations**: CR, cruciate ligament rupture; VAS, visual analogue scale score.

**Table 5:** Correlation between MR imaging quantification of cranial cruciate ligament properties and arthroscopic and histologic assessment.

	CrCL FSE Volume		CrCL FSE Grayscale		CrCL VIPR Volume		CrCL VIPR Grayscale		CrCL T1 Enhance	
	S_R_	*P value*	S_R_	*P value*	S_R_	*P value*	S_R_	*P value*	S_R_	*P value*
**Complete CR Stifle**										
**Histologic Synovitis Grade**	-0.08	0.64	-0.03	0.86	n/a		n/a		n/a	
**Histologic Synovitis VAS**	0.07	0.71	-0.02	0.91	n/a		n/a		n/a	
**Partial CR Stifle**										
**Histologic Synovitis Grade**	0.10	0.60	*0*.*50*	*0*.*007*	0.02	0.93	0.36	0.12	-0.28	0.14
**Histologic Synovitis VAS**	0.05	0.81	*0*.*50*	*0*.*006*	-0.21	0.37	0.32	0.17	*-0*.*43*	*0*.*02*
**Arthroscopic Fiber Damage VAS**	0.05	0.81	0.34	0.08	0.05	0.84	0.11	0.62	-0.21	0.27

**Note**: Arthroscopic fiber damage VAS assesses ligament fiber rupture in the cranial cruciate ligament in the partial CR stifle. FSE and VIPR grayscale values were normalized to the cranial tibial muscle. FSE and VIPR volume measurements were normalized to patellar length. n = 28 dogs for all CrCL FSE and T1 Enhance comparisons; n = 20 for all CrCL VIPR comparisons. **Abbreviations**: CR, cruciate ligament rupture; CrCL, cranial cruciate ligament; FSE, fast spin echo; VIPR, Vastly under-sampled Isotropic Projection; VAS, visual analogue scale score.

**Table 6:** Correlation between histologic assessment of synovial inflammatory cell populations and arthroscopic assessment of cranial cruciate ligament fiber damage.

	Histologic Synovitis VAS		Suppurative Inflammation Grade		CD3^+^ T-Lymphocyte Grade		TRAP^+^ Macrophage Grade		Factor VIII^+^ Vessel Grade		Synovial Factor VIII^+^ Vessel VAS	
	S_R_	*P value*	S_R_	*P value*	S_R_	*P value*	S_R_	*P value*	S_R_	*P value*	S_R_	*P value*
**Complete CR Stifle**												
**Histologic Synovitis Grade**	*0*.*62*	*0*.*0003*	0.02	0.91	0.20	0.31	0.06	0.74	0.05	0.80	0.03	0.88
**Histologic Synovitis VAS**	n/a		0.16	0.41	0.16	0.40	0.05	0.78	0.20	0.31	0.08	0.68
**Partial CR Stifle**												
**Histologic Synovitis Grade**	*0*.*79*	<0.0001	0.07	0.72	*0*.*38*	*0*.*05*	*0*.*46*	*0*.*01*	0.16	0.41	0.04	0.83
**Histologic Synovitis VAS**	n/a		0.002	0.99	0.30	0.13	*0*.*50*	*0*.*007*	0.32	0.11	0.29	0.14
**Arthroscopic Fiber Damage VAS**	*0*.*38*	*0*.*04*	0.02	0.91	0.35	0.07	0.16	0.40	0.34	0.09	0.36	0.06

**Note**: Arthroscopic fiber damage VAS assesses ligament fiber rupture in the cranial cruciate ligament in the partial CR stifle. n = 28–29 dogs. **Abbreviations**: CR, cruciate ligament rupture; CrCL, cranial cruciate ligament; VAS, visual analog scale score.

**Table 7:** Correlation between serum and synovial markers of inflammation, Histologic Synovitis Grade and cranial cruciate ligament fiber damage.

	Serum CRP		Serum ICTP		Synovial CRP		Synovial:Serum CRP		Synovial ICTP		Synovial:Serum ICTP		Stifle TNCC	
	S_R_	*P value*	S_R_	*P value*	S_R_	*P value*	S_R_	*P value*	S_R_	*P value*	S_R_	*P value*	S_R_	*P value*
**Complete CR Stifle**														
**Histologic Synovitis Grade**	0.32	0.09	-0.36	0.06	*0*.*44*	*0*.*02*	0.30	0.11	-0.16	0.43	0.36	0.06	-0.07	0.73
**Histologic Synovitis VAS**	0.35	0.06	-0.31	0.10	*0*.*44*	*0*.*02*	0.13	0.49	-0.22	0.25	0.31	0.10	-0.15	0.45
**Partial CR Stifle**														
**Histologic Synovitis Grade**	0.12	0.52	0.07	0.72	0.33	0.09	*0*.*42*	*0*.*03*	-0.02	0.94	-0.003	0.99	-0.06	0.77
**Histologic Synovitis VAS**	0.00	0.99	0.01	0.97	0.30	0.12	*0*.*48*	*0*.*01*	-0.30	0.13	-0.03	0.89	-0.09	0.65
**Arthroscopic Fiber Damage VAS**	0.07	0.73	-0.06	0.75	0.11	0.59	0.01	0.95	-0.02	0.93	0.16	0.43	0.18	0.37

**Note**: Arthroscopic fiber damage VAS assesses ligament fiber rupture in the cranial cruciate ligament in the partial CR stifle. n = 26–29 dogs. **Abbreviations**: CR, cruciate ligament rupture; CrCL, cranial cruciate ligament; CRP, C-reactive protein; ICTP, synovial pyridinoline cross-lined carboxy-terminal telopeptide of type I collagen; TNCC, total nucleated cell count.

**Fig 4:**
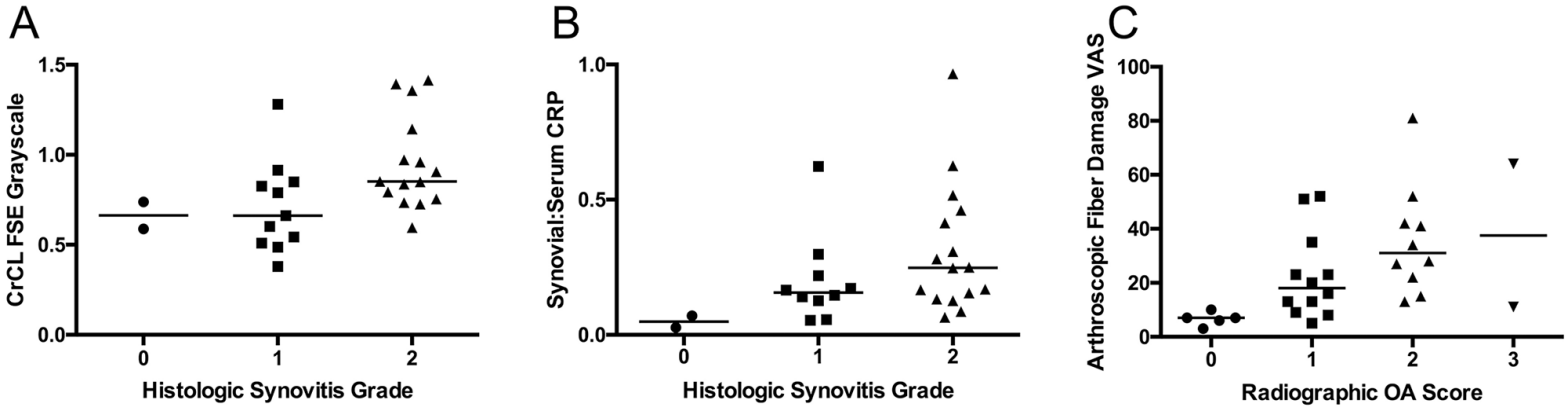
Relationship between partial cruciate rupture (CR) comparators and select variables. (A) Histologic Synovitis Grade was significantly correlated with cranial cruciate ligament (CrCL) fluid content, as reflected by CrCL FSE Grayscale value (*P* = 0.007, S_R_ = 0.50). (B) Histologic Synovitis Grade in the partial CR stifles was significantly associated with Synovial:Serum C-reactive protein (CRP) ratio (*P* = 0.03, S_R_ = 0.42), suggesting that CRP may be a biochemical marker for synovial inflammation. (C) Radiographic osteoarthritis (OA) Score was significantly correlated with Arthroscopic Fiber Damage visual analog scale (VAS) score (*P* = 0.0005, S_R_ = 0.61), supporting the use of radiography as a diagnostic test that reflects CR disease progression. The median values are indicated with a horizontal line.

For partial CR stifles (Fig 3B), the following factors were significantly correlated with Histologic Synovitis Grade: Arthroscopic Synovitis Score (S_R_ = 0.43, *P* = 0.02), CrCL FSE Grayscale (S_R_ = 0.50, *P* = 0.007, Fig 4A), Histologic Synovitis VAS (S_R_ = 0.79, *P*<0.0001), CD3^+^ T lymphocyte Grade (S_R_ = 0.38, *P* = 0.05), TRAP^+^ Macrophage Grade (S_R_ = 0.46, *P* = 0.015), and Synovial:Serum CRP (S_R_ = 0.42, *P* = 0.027, Fig 4B). Histologic Synovitis Grade was weakly correlated with the following factors: Synovial CRP (S_R_ = 0.33, *P* = 0.09).

For both partial CR and complete CR stifles, the variables used to calculate Histologic Synovitis Grade (Lymphocytic-Plasmacytic Inflammation, Synoviocyte Thickness and Synoviocyte Hypertrophy) had correlations with other variables similar to that of Histologic Synovitis Grade (S2–S6 Tables).

#### Correlations with Histologic Synovitis Visual Analogue Scale (VAS)

For complete CR stifles (Fig 3A), the following factors were significantly associated with Histologic Synovitis VAS: Synovial CRP (S_R_ = 0.44, *P* = 0.02), and Histologic Synovitis Grade (S_R_ = 0.62, *P* = 0.0003). Histologic Synovitis VAS was weakly correlated with the following factors: Serum CRP (S_R_ = 0.35, *P* = 0.06) and Synovial:Serum ICTP (S_R_ = 0.31, *P* = 0.10).

For partial CR stifles (Fig 3B), the following factors were significantly correlated with Histologic Synovitis VAS: Radiographic Effusion (S_R_ = 0.38, *P* = 0.04), Radiographic OA (S_R_ = 0.47, *P* = 0.01), Arthroscopic CrCL Fiber Damage VAS (S_R_ = 0.38, *P* = 0.04), CrCL FSE Grayscale (S_R_ = 0.50, *P* = 0.006), CrCL T1 Enhance (S_R_ = -0.43, *P* = 0.02), Histologic Synovitis Grade (S_R_ = 0.79, *P*<0.0001), TRAP^+^ Macrophage Grade (S_R_ = 0.50, *P* = 0.007), and Synovial:Serum CRP (S_R_ = 0.48, *P* = 0.009). Notably, eleven dogs had Synovial:Serum CRP above 0.2 in the partial CR stifle, suggesting elevated risk of subsequent contralateral rupture [32].

#### Correlations with Arthroscopic Fiber Damage

For partial CR stifles (Fig 3B), the following factors were significantly correlated with Arthroscopic Fiber Damage VAS: Radiographic OA (S_R_ = 0.60, *P* = 0.0005, Fig 4C), Arthroscopic Synovitis Score (S_R_ = 0.41, *P* = 0.028), Arthroscopic Synovitis VAS (S_R_ = 0.46, *P* = 0.01), and Histologic Synovitis VAS (S_R_ = 0.38, *P* = 0.04). Arthroscopic Fiber Damage VAS was weakly correlated with the following factors: CrCL FSE Grayscale (S_R_ = 0.34, *P* = 0.08), CD3^+^ T Lymphocyte Grade (S_R_ = 0.35, *P* = 0.07), Factor VIII^+^ Vessel Grade (S_R_ = 0.34, *P* = 0.09) and Factor VIII^+^ Vessel VAS (S_R_ = 0.36, *P* = 0.06) (Fig 2).

#### Correlation clustering

When the overall pattern of correlations was examined, data formed several clusters (Fig 3). In the complete CR stifle (Fig 3A), Synovial and Serum CRP levels correlated with several histologic markers of inflammation (Histologic Grade, Histologic VAS score, Factor VIII^+^ Vessel VAS score, Factor VIII^+^ Vessel Grade). In a second cluster, radiographic variables of inflammation (Radiographic Effusion and Radiographic OA) were correlated with biochemical markers (Synovial:Serum CRP and Stifle TNCC) and arthroscopic measures of joint inflammation (Total Arthroscopic Score and Arthroscopic VAS score). A third cluster highlighted positive correlations between CD3^+^ T lymphocytes, TRAP^+^ macrophages and suppurative inflammation.

In the partial CR stifle, a larger number of positive correlations were identified (Fig 3B). Suppurative inflammation was positively correlated with CrCL ligament volumes assessed by MR imaging (CrCL FSE Volume, CrCL VIRP Volume) and functional length of the ligament (CrCL_d_). In a second cluster, serum and stifle joint CRP concentrations were positively correlated with Stifle TNCC. In a third cluster, Synovial:Serum CRP was positively correlated with several histologic markers of inflammation, including Histologic Grade, Histologic Synovitis VAS score, TRAP^+^ Macrophage Grade, and CD3^+^ T Lymphocyte Grade. In a fourth cluster, arthroscopic variables (Arthroscopic CrCL Fiber Damage VAS score, Total Arthroscopic Score, Arthroscopic Synovitis VAS score) were positively correlated with both MR imaging ligament grayscale values (CrCL FSE Grayscale, CrCL VIPR Grayscale), Radiographic OA, and histologic Factor VIII^+^ variables (Factor VIII^+^ Vessel VAS score, Factor VIII^+^ Vessel Grade).

## Discussion

Canine CR is an acquired, progressive, complex disease with genetic and environmental risk factors. Risk of a second contralateral CR is related to the presence and severity of stifle synovitis [16] and to radiographic severity of stifle synovial effusion and osteophytosis in partial CR stifles at diagnosis [1]. MR imaging is often performed for evaluation of the human knee, but limited data are available for the canine stifle. A comprehensive assessment of relationships between radiographic and MR imaging and development of CrCL fiber damage in dogs is lacking. The present study aimed to evaluate the correlation between arthroscopic findings, histologic assessment, radiographic findings, inflammatory markers and MR imaging in dogs with unilateral complete CR and contralateral partial CR [1,5]. Importantly, in partial CR stifles, we found MR signal intensity in the CrCL was correlated with histologic synovitis (Histologic Synovitis Grade). We also found that radiographic OA was correlated with CrCL fiber damage in partial CR stifles. Few significant correlations were found in complete CR stifles, compared with partial CR stifles, suggesting that joint instability after complete ligament rupture has profound effects on the joint environment.

In dogs with unilateral complete CR, the risk for contralateral CR ranges from 22–54% at 6 to 17 months after diagnosis [33–37]. Stifle synovitis is associated with partial fiber rupture in the cruciate ligament complex and an increased risk of subsequent contralateral CR [3,16]. The relationship between the radiographic stifle effusion and OA in dogs with unilateral complete CR and contralateral partial CR provides a means to identify patients with an increased risk of contralateral CR [1,3,5–7]. In the present study, we found that radiographic OA was highly correlated with CrCL fiber damage in partial CR stifles.

Signalment in the present study is typical for the CR condition. Disease risk can be influenced by neuter status [38], increasing age [39], and breed. Breeds such as the Newfoundland, Rottweiler, Labrador Retriever, Bulldog and Boxer have a relatively high disease risk [39]. The use of NSAID medication before enrollment was variable. Little is known regarding the effect of NSAID treatment on stifle synovitis in dogs with CR, although it has been suggested that chronic NSAID administration may lead to a reduction in biomarkers of joint inflammation, such as synovial prostaglandin E2 [40]. Although the use of such medications may influence the degree of synovitis in the CR condition with long-term administration, it would not be expected to alter the correlations assessed in the present study.

The distribution of radiographic scores for osteophytosis and synovial effusion was similar to our earlier work [1], including increased effusion and osteophytosis in complete CR stifles. For partial CR stifles, we found that radiographic effusion and OA were correlated with synovitis severity; these variables have been significantly correlated in earlier work [6]. Interestingly, radiographic OA in partial CR stifles was strongly correlated with CrCL fiber rupture assessed arthroscopically. Chronicity of disease likely influences development of CrCL fiber damage, stifle inflammation and radiographic OA, as well as the their interrelationships, as the natural history of the disease progresses [8].

MR imaging is a preferred method for diagnosis of meniscal damage and anterior cruciate ligament rupture in human beings, compared with arthroscopy [41]. MR image contrast is typically T1- or T2-weighted, each of which highlights different anatomic structures. Intermediate weight sequences are preferred for musculoskeletal imaging. Three-dimensional MR imaging sequences are considered superior to 2D imaging due to a higher signal to noise ratio and the ability to acquire thin continuous slices [42]. To date, use of MR imaging in dogs with CR has focused on diagnosis of meniscal damage, with varying results regarding its utility [43–45]. In large animal models, MR imaging reflects CrCL graft properties; graft volume and median grayscale values are predictive of structural properties [18,19]. Contrast enhanced T1 weighted MR imaging of synovium also reflects stifle synovitis [21]. We used a traditional T1-weighted MR imaging sequence as well as two 3D intermediate weight sequences, 3D FSE Cube and VIPR-aTR. Both 3D FSE Cube and the VIPR-aTR provide high-resolution, fat suppressed images. The FSE Cube sequence also has a shorter acquisition time [46]. The VIPR-aTR sequence yields high resolution 3D reconstruction of ligament tissue [47]. In healthy CrCL, CrCL volume, as determined by either 3D FSE Cube or VIPR-aTR reconstruction, is correlated to structural properties *ex vivo* [20]. We normalized FSE Cube and VIPR-aTR CrCL grayscale values to the cranial tibial muscle to minimize any dog-specific or scan-specific variance [20]. Ligament volume was normalized to patellar length to help account for patient heterogeneity.

In stifles with partial CR, we found that CrCL FSE Grayscale was significantly correlated with histologic synovitis; a weaker correlation with arthroscopic CrCL fiber damage was also found. These observations likely reflect the anatomic relationship between the CrCL matrix and the overlying synovium. Increased water content has been reported in CrCL tissue from dogs with CR, compared to normal dogs [48], and we found a significant decrease in FSE Cube CrCL signal intensity in the partial CR stifle compared to the complete CR stifle, indicating that the complete CR CrCL tissue had increased fluid content. Increased CrCL MR signal intensity using proton-density-weighted sagittal sequences has also been identified in dogs with CR [43], although contralateral stable stifles were not evaluated. Taken together, these findings suggest that changes in CrCL MR signal intensity indicates varying degrees of fluid accumulation within the ligament tissue, likely reflecting ligament matrix fiber damage and associated edema and inflammation in the overlying synovium. The synovial sheath surrounding the CrCL has a functionally important but poorly understood role in CrCL homeostasis that includes a protective barrier function with regard to the surrounding stifle joint synovial fluid [49,50]. The ligament matrix physiological environment is influenced by surrounding synovial fluid because of a blood-ligament barrier in the CrCL microvasculature [49]. Vascular density in CrCL from dogs with CR is increased when compared to normal CrCL tissue [51]. Interestingly, although overall synovitis severity was increased in the complete CR stifles, synovial TNCC was increased in partial CR stifles, suggesting that further investigation of synovial fluid cell phenotypes in the early phase of the disease is needed to better understand the role of specific cell types in progressive ligament failure.

In normal dogs *ex vivo*, estimation of CrCL ligament volume using MR imaging correlates with ligament structural properties, including yield load and load to failure [20]. In the present study, normalized CrCL ligament volume, estimated using both FSE Cube and VIPR-aTR sequences, did not correlate with synovitis or CrCL fiber damage in the partial CR stifle. We found significant loss of FSE Cube CrCL volume in the complete CR stifle compared with the partial CR stifle. Collectively, these findings suggest that substantial ligament resorption and loss of MR imaging volume occurs after complete rupture and development of joint instability. Notably, the four dogs with very mild instability in the partial CR stifle indicative of Grade II CrCL sprain had CrCL volumes that were similar to the other study dogs. Defining the relationship between a specific sprain grade and MR imaging volume measurements would require more detailed assessment of structural properties.

Synovitis is an important factor influencing progressive CrCL fiber rupture [6]. Joints were evaluated using total histologic synovitis grade [7], taking into account synovial hypertrophy, vascularity, and synovitis in 6 compartments within each stifle. Histologic synovitis was not significantly different between partial and complete CR stifles, suggesting that inflammation is well established at an early phase of the CR disease process. In the partial CR stifles, arthroscopic synovitis was significantly correlated with both histologic synovitis, as previously reported [6], and arthroscopic CrCL damage. Arthroscopic synovial vascularity in partial CR stifles significantly influences risk of disease progression to complete CR [7]. These results support the concept that stifle synovitis has significant deleterious effects on CrCL structural properties [23].

Synovial cell populations in partial CR stifles includes populations of T and B lymphocytes, TRAP^+^ macrophages, major histocompatibility complex (MHC) class II^+^ dendritic cells, and plasma cells [7,52–54]. Our histologic grading principally assessed infiltration of the intimal and subintimal tissues with inflammatory cells, and did not account for distribution of specific inflammatory cell types. T lymphocytes are an important component of the inflammatory cell population found in synovium of dogs with CR [3,7,53]. In partial CR stifles, synovial CD3^+^ T lymphocytes correlated moderately with histologic synovitis and arthroscopic CrCL fiber damage; this correlation was not found in complete CR stifles. These findings suggest that the lymphocytic synovitis develops in the early phase of the CR condition. Synovial CD3^+^ T lymphocyte numbers were higher in complete CR stifles, reflecting the greater degree of synovial inflammation in these stifles. While T lymphocytes are generally present in chronically inflamed tissue, neutrophilic infiltration reflects a more acute inflammatory process; we found only mild suppurative inflammation in the stifles in this study.

Activated TRAP^+^ macrophages are not found in normal synovium, but are often found in the synovium of dogs with CR [7,54]. In partial CR stifles, we found the population of TRAP^+^ macrophages correlated with histologic synovitis. Numbers of TRAP^+^ synovial macrophages were also increased in complete CR stifles compared with partial CR stifles. Activated macrophages play an important role in the development of OA pathology in mouse models [55,56] and likely influence matrix degradation within the cruciate ligament complex. Macrophages have distinct polarization states that are associated with physiological function [57]. Further investigation into macrophage phenotypes in dogs with CR is warranted.

Factor VIII^+^ vessel staining was performed as a marker for synovial angiogenesis. Synovial histologic vascularity was higher in synovium from complete CR stifles, compared with partial CR stifles. In partial CR stifles, we found mild correlation between Factor VIII^+^ staining and arthroscopic CrCL fiber damage, suggesting synovial vascularity promotes disturbance to ligament matrix homeostasis. In earlier work, we found Factor VIII^+^ vascularity was significantly correlated with arthroscopic vascularity and synovitis [7]. Overall, these observations suggest that angiogenesis within stifle synovium is an important component of CR pathogenesis.

CRP is an acute-phase protein which is elevated with development of inflammation. In humans with OA, serum CRP is typically 3,000 to 8,000 μg/L [58]. Several dogs in this study had serum CRP values >8,000 μg/L, indicating that a severe systemic inflammatory response may be associated with the CR disease; CRP may be a marker for risk of disease progression [59]. Synovial:Serum CRP was correlated with histologic synovitis in partial CR stifles. Synovial CRP was correlated with histologic synovitis in the complete CR stifles. Overall, these results support the concept that serum and synovial CRP reflects severity and progression of OA [60,61]. Recent research suggests that a synovial:serum ratio greater than 0.2 is a risk factor for subsequent contralateral CR [32]. Further work evaluating the potential of CRP as a marker for risk of complete CR is needed.

ICTP reflects increased matrix metalloproteinase-mediated collagen degradation in human patients with rheumatoid arthritis (RA) [62]. As a biomarker of collagen metabolism, ICTP concentrations in serum and synovial fluid can be used to assess disease severity in human rheumatoid arthritis patients [63]. In the present study, synovial ICTP levels were not significantly different between the complete and partial CR stifles. A weak positive correlation between synovial:serum ICTP and histologic synovitis in the complete CR stifle was identified, suggesting ICTP may reflect ligament collagen breakdown, although the weak negative correlation with serum ICTP confounds this observation. This suggests that ICTP is unlikely to be a useful biomarker for CrCL matrix breakdown in dogs with CR.

A strength of this study is also a limitation, in that we studied a clinically-relevant heterogeneous population of client-owned dogs. Variations in patient history, duration of disease, and use of NSAID medications may have influenced the results. Histologic Synovitis Grade and Histologic Synovitis VAS were determined from synovial joint biopsies from the medial joint compartment, which may not be reflective of overall joint synovial inflammation, although regional differences in synovitis were not identified in earlier work [7]. Variation in arthroscopic grading may also have influenced our results. 3.0 Tesla MR imaging scans were only obtained in 28 of the 29 dogs. The first dog enrolled in the study had an MR imaging scan performed with a 1.5T MR imaging machine; the images from this study were not deemed acceptable and henceforth a 3T MR imaging was used. The VIPR-aTR sequence was not obtained for all dogs due to limited availability.

This study is the first to evaluate correlations between multiple diagnostic measures for dogs with unilateral complete CR and contralateral partial CR. We chose Histologic Synovitis Grade and Histologic Synovitis VAS score in both the partial CR and complete CR stifles and Arthroscopic Fiber Damage VAS in the partial CR stifles as comparators. We found that MR FSE Cube grayscale quantification was a marker for stifle synovitis and may provide a means of assessing CrCL degradation in partial CR stifles. We also found that radiographic OA was correlated with CrCL fiber damage in partial CR stifles. Additionally, we found that CRP levels in serum and synovial fluid reflect severity of synovitis in both the partial CR and complete CR stifles. Collectively, this work identifies several clinically relevant diagnostic markers that could be used for stifle assessment in longitudinal studies of dogs with CR.

## Supporting information

S1 Table List of variables. (DOCX)

S2 Table Correlation between radiographic measures and components of histologic grade. (DOCX)

S3 Table Correlation between arthroscopic synovitis and components of histologic grade. (DOCX)

S4 Table Correlation between MR imaging quantification and components of histologic grade. (DOCX)

S5 Table Correlation between histologic assessment of synovial inflammatory cell populations and components of histologic grade. (DOCX)

S6 Table Correlation between serum and synovial markers of inflammation and components of histologic grade. (DOCX)

S1 File Study data is provided in the supporting excel file PONE-S-16-43023 data. (XLSX)
