# Supplementary material for: Radiographic and magnetic resonance imaging predicts severity of cruciate ligament fiber damage and synovitis in dogs with cranial cruciate ligament rupture
Source: PLoS One. 2017 Jun 2;12(6):e0178086. doi: 10.1371/journal.pone.0178086 (PMC5456057; doi:10.1371/journal.pone.0178086)
Supplement: S1 Table — (DOCX) [file pone.0178086.s001.docx]

**S1 Table**. List of variables.

| Radiographic Effusion | Radiographic stifle joint effusion |
| --- | --- |
| Radiographic OA | Radiographic stifle osteoarthritis |
| TPA | Tibial plateau angle, determined from a lateral radiographic view of the stifle, with the stifle and tarsus held in ninety degrees of flexion. |
| CrCL_d_ | Cranial cruciate ligament length normalized to the patellar length, both measured from a lateral radiograph |
| CrCL FSE Volume | Cranial cruciate ligament volume, measured from MR imaging using the 3D fast spin echo Cube sequence, normalized to the patellar length, determined using a lateral radiograph |
| CrCL FSE Grayscale | Cranial cruciate ligament greyscale value, normalized to the cranial tibial muscle grayscale value, measured from MR imaging using the 3D fast spin echo Cube sequence |
| CrCL VIPR Volume | Cranial cruciate ligament volume measured from MR imaging using the Vastly under-sampled Isotropic PRojection with alternating length repetition times sequence, normalized to the patellar length, determined using a lateral radiograph |
| CrCL VIPR Grayscale | Cranial cruciate ligament grayscale value, normalized to the cranial tibial muscle grayscale value, measured from MR imaging using the Vastly under-sampled Isotropic PRojection with alternating length repetition times sequence |
| CrCL T1 Enhance | The change in cranial cruciate ligament grayscale values measured before and after intravenous gadolinium, using T1-weighted sequences. Both pre- and post-contrast grayscale values were normalized to the pre-contrast median grayscale value of the cranial tibial muscle. |
| Total Arthroscopic Score | Using a standardized scoring system, Total Arthroscopic Score was calculated as the sum of three parameters (synovial hypertrophy, vascularity, and synovitis) grade, measured in 6 joint compartments (lateral and medial pouches, lateral and medial femoro-tibial joint compartments, the intercondylar notch, and the femoro-patellar joint). |
| Arthroscopic Synovitis VAS | Arthroscopic Synovitis Visual Analog Scale score. The degree of global synovitis was scored using a visual analog scale (0-100) for each stifle joint, with 0 representing no inflammation, and 100 signifying the most severe inflammation |
| Arthroscopic CrCL Fiber Damage VAS | Arthroscopic Cranial Cruciate Ligament Fiber Damage Visual Analog Scale score. The extent of CrCL fiber damage was determined during arthroscopic evaluation, using a visual analog scale (1-100), with 0 representing no damage, and 100 signifying complete ligament fiber rupture. |
| Histologic Synovitis Grade | H&E stained synovial biopsies were scored for lymphocytic-plasmacytic and suppurative inflammation of the synovial intima, synovial cell hypertrophy, synovial intima width seen at the center of five random high power fields. The sum of these scores were used to assign the Histologic Synovitis Grade. |
| Histologic Synovitis VAS | Histologic Synovitis Visual Analog Scale score. Overall synovitis from H&E stained synovial biopsies, scored using a visual analog scale (1-100) with 0 representing no inflammation and 100 signifying the most severe inflammation (100). |
| Suppurative Inflammation | H&E stained synovial biopsies were scored on a numeric scale for neutrophilic infiltration. This variable was used as a component for the determination of Histologic Synovitis Grade. |
| Lymphocytic-Plasmacytic Inflammation | H&E stained synovial biopsies were scored on a numeric scale for lymphocyte and plasma cell infiltration. This variable was used as a component for the determination of Histologic Synovitis Grade. |
| Synoviocyte Thickness | H&E stained synovial biopsies were scored on a numeric scale for the number of synoviocyte cell layers. This variable was used as a component for the determination of Histologic Synovitis Grade. |
| Synoviocyte Hypertrophy | H&E stained synovial biopsies were scored on a numeric scale for the degree of synoviocyte hypertrophy and density. This variable was used as a component for the determination of Histologic Synovitis Grade. |
| CD3^+^ T Lymphocyte Grade | The amount of CD3^+^ T Lymphocytes in each synovial biopsy was graded on a numeric scale. |
| TRAP^+^ Macrophage Grade | The amount of TRAP^+^ Macrophages in each synovial biopsy was graded on a numeric scale. |
| Factor VIII^+^ Vessel Grade | The amount of Factor VIII^+^ Vessels in each synovial biopsy was graded on a numeric scale. |
| Factor VIII^+^ Vessel VAS | Factor VIII^+^ Vessel Visual Analog Scale score. Overall Factor VIII+ vessel staining was scored using a visual analog scale (1-100) with 0 representing no positive stain and 100 signifying the most severe stain update (100). |
| Serum CRP | Serum C-Reactive Protein concentration |
| Serum ICTP | Serum pyridinoline cross-lined carboxy-terminal telopeptide of type I collagen concentration |
| Synovial CRP | Synovial C-Reactive Protein concentration |
| Synovial ICTP | Synovial pyridinoline cross-lined carboxy-terminal telopeptide of type I collagen concentration |
| Synovial:Serum CRP | Synovial to Serum C-Reactive Protein Ratio. |
| Synovial:Serum ICTP | Synovial to Serum pyridinoline cross-lined carboxy-terminal telopeptide of type I collagen Ratio |
| Stifle TNCC | Stifle Total Nucleated Cell Count. An estimation of total nucleated cell counts estimated using direct smears of synovial fluid, using a validated estimation method. |
