# Supplementary material for: Radiographic and magnetic resonance imaging predicts severity of cruciate ligament fiber damage and synovitis in dogs with cranial cruciate ligament rupture
Source: PLoS One. 2017 Jun 2;12(6):e0178086. doi: 10.1371/journal.pone.0178086 (PMC5456057; doi:10.1371/journal.pone.0178086)
Supplement: S2 Table — (DOCX) [file pone.0178086.s002.docx]

**S2 Table**. Correlation between radiographic measures and components of histologic grade

|  | **Radiographic Effusion** | | **Radiographic OA** | | **CrCL_D_** | |
| --- | --- | --- | --- | --- | --- | --- |
|  | S_R_ | *P value* | S_R_ | *P value* | S_R_ | *P value* |
| **Complete CR Stifle** | | | | | | |
| **Lymphocytic-Plasmacytic Inflammation** | -0.26 | 0.17 | -0.02 | 0.91 | -0.09 | 0.64 |
| **Synoviocyte Thickness** | 0.12 | 0.55 | -0.23 | 0.23 | -0.07 | 0.70 |
| **Synoviocyte Hypertrophy** | 0.24 | 0.21 | -0.21 | 0.28 | -0.04 | 0.81 |
| **Partial CR Stifle** | | | | | | |
| **Lymphocytic-Plasmacytic Inflammation** | *0.36* | *0.05* | 0.35 | 0.06 | -0.12 | 0.51 |
| **Synoviocyte Thickness** | *0.45* | *0.01* | 0.28 | 0.13 | -0.20 | 0.29 |
| **Synoviocyte Hypertrophy** | 0.22 | 0.24 | 0.26 | 0.18 | -0.10 | 0.61 |

**Abbreviations**; CR – cruciate ligament rupture; CrCL – cranial cruciate ligament. CrCL_D_ - radiographic length of the CrCL normalized to patellar length; n=29 dogs.
