# Supplementary material for: Radiographic and magnetic resonance imaging predicts severity of cruciate ligament fiber damage and synovitis in dogs with cranial cruciate ligament rupture
Source: PLoS One. 2017 Jun 2;12(6):e0178086. doi: 10.1371/journal.pone.0178086 (PMC5456057; doi:10.1371/journal.pone.0178086)
Supplement: S3 Table — (DOCX) [file pone.0178086.s003.docx]

**S3 Table**. Correlation between arthroscopic synovitis and components of histologic grade.

|  | **Arthroscopic Synovitis Score** | | **Arthroscopic Synovitis VAS** | | **Arthroscopic Fiber Damage VAS** | |
| --- | --- | --- | --- | --- | --- | --- |
|  | S_R_ | *P value* | S_R_ | *P value* | S_R_ | *P value* |
| **Complete CR Stifle** | | | | | | |
| **Lymphocytic-Plasmacytic Inflammation** | 0.18 | 0.36 | 0.22 | 0.25 | n/a | |
| **Synoviocyte Thickness** | -0.27 | 0.15 | -0.31 | 0.09 | n/a | |
| **Synoviocyte Hypertrophy** | -0.12 | 0.58 | -0.13 | 0.49 | n/a | |
| **Partial CR Stifle** | | | | | | |
| **Lymphocytic-Plasmacytic Inflammation** | 0.20 | 0.30 | 0.18 | 0.05 | 0.29 | 0.12 |
| **Synoviocyte Thickness** | 0.15 | 0.42 | 0.25 | 0.19 | 0.19 | 0.31 |
| **Synoviocyte Hypertrophy** | *0.47* | *0.01* | *0.38* | *0.04* | 0.33 | 0.08 |

**Note**: Arthroscopic fiber damage VAS assess ligament fiber rupture in the cranial cruciate ligament in the partial CR stifle. n=29 dogs. **Abbreviations**: CR, cruciate ligament rupture; VAS, visual analogue scale score.
