# Supplementary material for: Radiographic and magnetic resonance imaging predicts severity of cruciate ligament fiber damage and synovitis in dogs with cranial cruciate ligament rupture
Source: PLoS One. 2017 Jun 2;12(6):e0178086. doi: 10.1371/journal.pone.0178086 (PMC5456057; doi:10.1371/journal.pone.0178086)
Supplement: S4 Table — (DOCX) [file pone.0178086.s004.docx]

**S4 Table.** Correlation between MR imaging quantification and components of histologic grade

|  | **CrCL FSE Volume** | | **CrCL FSE Greyscale** | | **CrCL VIPR Volume** | | **CrCL VIPR Greyscale** | | **CrCL T1 Enhance** | |
| --- | --- | --- | --- | --- | --- | --- | --- | --- | --- | --- |
|  | S_R_ | *P value* | S_R_ | *P value* | S_R_ | *P value* | S_R_ | *P value* | S_R_ | *P value* |
| **Complete CR Stifle** | | | | | | | | | | |
| **Lymphocytic-Plasmacytic Inflammation** | 0.04 | 0.85 | -0.04 | 0.83 | n/a | | n/a | | n/a | |
| **Synoviocyte Thickness** | 0.11 | 0.55 | -0.06 | 0.77 | n/a | | n/a | | n/a | |
| **Synoviocyte Hypertrophy** | -0.23 | 0.25 | -0.21 | 0.28 | n/a | | n/a | | n/a | |
| **Partial CR Stifle** | | | | | | | | | | |
| **Lymphocytic-Plasmacytic Inflammation** | 0.05 | 0.78 | *0.55* | *0.002* | -0.15 | 0.54 | *0.50* | *0.03* | -0.16 | 0.42 |
| **Synoviocyte Thickness** | 0.17 | 0.39 | 0.21 | 0.29 | 0 | 0.99 | 0.12 | 0.61 | -0.04 | 0.85 |
| **Synoviocyte Hypertrophy** | 0.15 | 0.46 | 0.19 | 0.34 | -0.21 | 0.37 | -0.08 | 0.72 | 0.05 | 0.80 |

**Note**: FSE and VIPR greyscale values were normalized to the cranial tibial muscle. FSE and VIPR volume measurements were normalized to patellar length. n=28 dogs for all CrCL FSE and T1 Enhance comparisons; n=20 for all CrCL VIPR comparisons. **Abbreviations**: CR, cruciate ligament rupture; CrCL, cranial cruciate ligament; FSE, Fast Spin Echo; VIPR, vastly undersampled Vastly under-sampled Isotropic Projection, VAS, visual analogue scale score.
