# Supplementary material for: Radiographic and magnetic resonance imaging predicts severity of cruciate ligament fiber damage and synovitis in dogs with cranial cruciate ligament rupture
Source: PLoS One. 2017 Jun 2;12(6):e0178086. doi: 10.1371/journal.pone.0178086 (PMC5456057; doi:10.1371/journal.pone.0178086)
Supplement: S5 Table — (DOCX) [file pone.0178086.s005.docx]

**S5 Table.** Correlation between histologic assessment of synovial inflammatory cell populations and components of histologic grade

|  | **Histologic Synovitis VAS** | | **Suppurative Inflammation Grade** | | **CD3^+^ T-Lymphocyte Grade** | | **TRAP^+^ Macrophage Grade** | | **Factor VIII^+^ Vessel Grade** | | **Synovial Factor VIII^+^ Vessel VAS** | |
| --- | --- | --- | --- | --- | --- | --- | --- | --- | --- | --- | --- | --- |
|  | S_R_ | *P value* | S_R_ | *P value* | S_R_ | *P value* | S_R_ | *P value* | S_R_ | *P value* | S_R_ | *P value* |
| **Complete CR Stifle** | | | | | | | | | | | | |
| **Lymphocytic-Plasmacytic Inflammation** | 0.79 | <0.0001 | 0.11 | 0.58 | 0.30 | 0.11 | 0.17 | 0.39 | 0.10 | 0.62 | -0.08 | 0.68 |
| **Synoviocyte Thickness** | 0.05 | 0.81 | *-0.44* | *0.02* | -0.03 | 0.89 | 0.04 | 0.86 | 0.11 | 0.58 | 0.31 | 0.10 |
| **Synoviocyte Hypertrophy** | 0.34 | 0.07 | -0.10 | 0.72 | 0.07 | 0.73 | 0.29 | 0.13 | 0.03 | 0.86 | 0.17 | 0.39 |
| **Partial CR Stifle** | | | | | | | | | | | | |
| **Lymphocytic-Plasmacytic Inflammation** | *0.93* | *<0.0001* | 0.03 | 0.89 | 0.14 | 0.48 | *0.52* | *0.005* | 0.25 | 0.20 | 0.20 | 0.31 |
| **Synoviocyte Thickness** | *0.59* | *0.0007* | -0.05 | 0.80 | *0.47* | *0.01* | 0.31 | 0.11 | 0.32 | 0.10 | 0.20 | 0.32 |
| **Synoviocyte Hypertrophy** | *0.58* | *0.0009* | -0.25 | 0.19 | *0.54* | *0.003* | *0.38* | *0.04* | 0.27 | 0.16 | 0.14 | 0.47 |

**Note**: n=28-29 dogs. **Abbreviations**: CR, cruciate ligament rupture; CrCL, cranial cruciate ligament; VAS, visual analog scale score.
