# Supplementary material for: Radiographic and magnetic resonance imaging predicts severity of cruciate ligament fiber damage and synovitis in dogs with cranial cruciate ligament rupture
Source: PLoS One. 2017 Jun 2;12(6):e0178086. doi: 10.1371/journal.pone.0178086 (PMC5456057; doi:10.1371/journal.pone.0178086)
Supplement: S6 Table — (DOCX) [file pone.0178086.s006.docx]

**S6 Table**. Correlation between serum and synovial markers of inflammation and components of histologic grade

|  | **Serum CRP** | | **Serum ICTP** | | **Synovial CRP** | | **Synovial: Serum CRP** | | **Synovial ICTP** | | **Synovial:Serum ICTP** | | **Stifle TNCC** | | |
| --- | --- | --- | --- | --- | --- | --- | --- | --- | --- | --- | --- | --- | --- | --- | --- |
|  | S_R_ | *P value* | S_R_ | *P value* | S_R_ | *P value* | S_R_ | *P value* | S_R_ | *P value* | S_R_ | *P value* | S_R_ | *P value* | |
| **Complete CR Stifle** | | | | | | | | | | | | | | | |
| **Lymphocytic-Plasmacytic Inflammation** | 0.29 | 0.12 | -0.17 | 0.37 | *0.37* | *0.05* | 0.11 | 0.56 | 0.03 | 0.87 | 0.19 | 0.34 | 0.08 | | 0.69 |
| **Synoviocyte Thickness** | 0.25 | 0.19 | -0.33 | 0.08 | 0.30 | 0.12 | 0.32 | 0.09 | 0.39 | 0.84 | *0.45* | *0.02* | -0.09 | | 0.65 |
| **Synoviocyte Hypertrophy** | 0.35 | 0.06 | -0.31 | 0.10 | *0.40* | *0.03* | 0.30 | 0.11 | -0.27 | 0.16 | 0.35 | 0.06 | -0.09 | | 0.65 |
| **Partial CR Stifle** | | | | | | | | | | | | | | | |
| **Lymphocytic-Plasmacytic Inflammation** | -0.67 | 0.73 | -0.04 | 0.84 | 0.26 | 0.18 | *0.52* | *0.004* | -0.22 | 0.28 | 0.06 | 0.76 | -0.18 | | 0.37 |
| **Synoviocyte Thickness** | 0.31 | 0.11 | -0.03 | 0.87 | *0.52* | *0.004* | *0.53* | *0.004* | 0.11 | 0.59 | 0.16 | 0.44 | 0.04 | | 0.84 |
| **Synoviocyte Hypertrophy** | 0.05 | 0.79 | -0.05 | 0.81 | 0.20 | 0.30 | *0.38* | *0.05* | 0.05 | 0.81 | 0.09 | 0.66 | 0.08 | | 0.67 |

**Note**. n=26-29 dogs. **Abbreviations**; CR, cruciate ligament rupture; CrCL, cranial cruciate ligament; CRP, C-reactive protein; ICTP, synovial pyridinoline cross-lined carboxy-terminal telopeptide of type I collagen; TNCC, total nucleated cell count.
